# Macular Surgery: Classification, Management and Surgical Techniques

**DOI:** 10.1155/2023/9891345

**Published:** 2023-01-16

**Authors:** Matteo Forlini, Stanislao Rizzo, Robert Rejdak, Sundaram Natarajan, Teresio Avitabile, Rodolfo Mastropasqua

**Affiliations:** ^1^San Marino Hospital, San Marino; ^2^Fondazione Policlinico Universitario Agostino Gemelli, Universita' Cattolica Del Sacro Cuore, Rome, Italy; ^3^Medical University in Lublin, Lublin, Poland; ^4^Aditya Jyot Eye Hospital, Mumbai, India; ^5^University of Catania, Catania, Italy; ^6^University of Modena, Modena, Italy

In recent years, there has been a rapid advance in diagnostic modalities as well as therapeutic interventions for macular diseases. Multimodal imaging enables a highly accurate classification of macular diseases which in turn permits us to follow a rational therapeutic approach. These macular diseases special issue focuses on the diagnostic approach and pathological classification as well as on the medical and surgical treatments available today.

The multimodal imaging (MMI) helps in accurate diagnosis and detailed characterization of macular hole, epiretinal membrane, and the vitreomacular interface syndromes [1–3].

The OCT-A has the potential of predicting prognosis of macular pathologies. The foveal avascular zone (FAZ) widening, changes in vessels density (VD), and perfusion density (PD) are altered after macular surgery and membrane peeling. Low values of these data correlate with poor visual and anatomical outcomes [4]. MMI permits close monitoring for spontaneous closure of small-diameter traumatic macular hole. The usual period for this may extend till 3 months. Surgical option may be considered beyond this period [5].

ILM peeling may inadvertently remove ganglion cells. This is generally confirmed by immunohistochemistry (IHC) of excised ILM tissue. The same can be confirmed *in vivo* by noting decreased GCC (ganglion cells complex) thickness on OCT scan [6]. This OCT finding points towards hazards of indiscriminate ILM peeling. Hence, one must gauge the benefit of ILM peeling against the visual risk of the same. In their meta-analysis, Ma et al. highlighted better visual outcomes and reduced complications with 27G ports in comparison to 25G vitrectomy, for the treatment of epiretinal membranes (ERMs) [7].

Furthermore, we can read how limited vitrectomy is a time-efficient and effective surgical procedure for removal of epiretinal membrane with no additional complications [8].

Historically, traumatic macular hole (TMH) was deemed to have poor visual prognosis and conservative management was the norm. It has now been shown that vitrectomy combined with ILM peeling achieves better anatomical closure and improved visual outcomes [5].

In patients with idiopathic full‐thickness macular hole (MHI  ≤0.5), a larger ILM peel of 4 disc diameters (DDs) appears to yield better anatomical outcomes than a limited 2 DD peel [9].

Furthermore, this special issue will focus on anatomical and functional outcomes of idiopathic macular hole revision surgery, after failed primary surgery. The success rate of revision surgery in eyes with unclosed MH was 85% and demonstrated an improvement in VA [10]. The possibilty to use autologous material to improve recovery in refractory MH is very interesting; usually autologous internal limiting membrane flap [11] appears to be effective in the closure of recurrent idiopathic macular holes. Also, we can see how autologous lens capsular flap transplantation can represent a potential alternative treatment for patients with large persistent macular holes after failure of other surgical techniques [12].

Innovations in macular surgery field also deals with use of various tamponades. The conscious choice of tamponade in each case can offer the best possible outcomes. Based on the OCT images, we can choose tamponade for each case; for MH ≤400 *μ*m, a high closure rate can be achieved by combining just air as a tamponade with prone position. However, for larger macular holes >400 *μ*m, the greatest anatomical success can be achieved by using little longer-acting SF6 tamponade in combination with the prone position [13].

This special issue also deals with use of pharmacological agents, like anti-VEGF. In patients with diabetic macular edema associated with vitreomacular interface abnormalities (VMAs), injections of ranibizumab have shown a better anatomical and functional improvement when compared with pars plana vitrectomy [14]. Further evidence shows how antiangiogenic agents, although experimental today, should be considered for persistent and refractory macular oedema [15]. Dexamethasone implant represents an effective treatment for postoperative macular oedema secondary to ERM and post-RD vitrectomy. It showed a significant improvement in anatomical as well as visual outcome. Intravitreal injections may represent a good option in diabetic patients with macular oedema associated with vitreomacular interface abnormalities [16, 17].

In conclusion, this special issue has a platter of original research articles and experimental studies, as well as case series on vitreoretinal interface disorders and macular surgery, illustrating and discussing functional and/or anatomical outcomes. This may offer readers a new perspective in dealing with macular pathologies and stimulate further research.

*Matteo Forlini* *Stanislao Rizzo* *Robert Rejdak* *Sundaram Natarajan* *Teresio Avitabile* *Rodolfo Mastropasqua*
